# The Story of the Insane from Year to Year

**Published:** 1901-07-27

**Authors:** 


### THE STORY OF THE INSANE FROM YEAR TO YEAR.

(THIRTEENTH SERIES.)

### THE ROYAL ASYLUM, PERTH.

THIS asylum contained 119 patients on January 1st, 1899, and 122 on the last day of the same year. There were 33 first admissions, 6 re-admissions, 8 recoveries, 15 discharged relieved, and 10 deaths. The average number resident was 120, and the total number of persons under care was 158. Calculated on the admissions the recoveries show a percentage of 20·51. This is, of course, a low recovery rate; but as we have remarked more than once in our notices of small asylums no definite conclusion can be draw from the statistics of any one year. Taking a number of years, the average recovery rate at the Perth Asylum works out at the creditable percentage of 38·95. The death rate for 1899 standing at 8·32 per cent. on the average number resident was also much above their average, which is only 5·67. Of these deaths 3 were caused by consumption, 2 by general paralysis, and 1 by epilepsy. Dr. Urquhart had not a very promising lot of admissions to work on during the year. Making every possible allowance, he says, not more than 16 could be looked on as curable cases; and we can readily understand this when we hear that 32 of the admissions had advanced degenerative disease, 20 had had previous attacks of insanity, and at the end of the year the asylum contained only 10 presumably curable cases. We should like to quote the last twenty-nine lines of Dr. Urquhart's report, but want of space forbids. He remarks that some people are sanguine enough to believe that the calamity of mental diseases has reached its maximum incidence; but he adds "there remains the fact that for the greater number of the insane there is no cure." He refers to the "Draconian methods of ancient Scotland for the prevention of the procreation of the unfit. . . . These methods offend us in their crude directness of application; but, nevertheless, it is towards prevention rather than cure that the nation must turn." It is true that these Draconian methods shock the delicate, chaste ears of the present generation, but at least these methods were successful, whereas our modern system of making post-mortem examinations by wholesale, looking through microscopes, and sticking photographs in case books, however desirable these points may be, will have no effect whatever in staying the progress of this terrible disease. Let anyone of our readers turn to the annual reports of our county asylums, and see what is therein stated as to the part played in the production of insanity by heredity and by drink—both preventible causes—and he would then see the full truth of Dr. Urquhart's remark that it is towards prevention and not cure that the nation must turn. Dr. Urquhart thinks the twentieth century may bear fruit. Well, we are not wholly without hope, and we should have more still if our asylum medical officers would leave their post-mortem rooms, even for a season, and begin to preach the new evangel. Anyway it is refreshing to find one medical superintendent who both sees it and says it. We have not always run on parallel lines with Dr. Urquhart, but we are glad to be able to do so in this instance.

### COUNTY OF LONDON ASYLUM AT BANSTEAD.

This asylum contained 2,435 patients on January 1, 1899, and on December 31 the number was 2,428. There were 358 first admissions, 83 readmissions, 170 recoveries, six discharged relieved, and 196 deaths. Calculated on the admissions the recoveries stand at 38·54 per cent., and calculated on the average number resident the percentage of deaths stands at 8·04 per cent. If transfers from other asylums be excluded when reckoning the percentage of recoveries, it would rise to 40·57. Of the year's deaths not fewer than 123 were caused by cerebral and spinal diseases, 24 by consumption, and 21 by pneumonia and bronchitis, the deaths from these two diseases being classed together. It would seem that 60 of the cases admitted in 1899 owed their insanity to such moral causes as domestic trouble, mental anxiety, and religion, while drink accounts for 75, hereditary tendency for 62, previous attacks for 95, and in 73 no cause was known. In 112 cases Dr. Shaw was able to obtain port-mortem examinations. Classes were held for the preparation of attendants and nurses for the certificate granted by the Medico-Psychological Association. We are not told how many presented themselves for this examination; but six male attendants passed it. Pensions were granted to the chaplain, Dr. Reade, to one of the farm labourers, to the shoemaker, to a stoker, and to a night-attendant. The committee express their appreciation of the work of Dr. Shaw and the staff generally. The weekly rate of maintenanee is 9s. 11d. per head—a rate considerably above the average.

